# Prospective associations between changes in physical activity and sedentary time and subsequent lean muscle mass in older English adults: the EPIC-Norfolk cohort study

**DOI:** 10.1186/s12966-023-01547-6

**Published:** 2024-01-26

**Authors:** Dharani Yerrakalva, Samantha Hajna, Kay-Tee Khaw, Simon J. Griffin, Soren Brage

**Affiliations:** 1 Department of Public Health and Primary Care, University of Cambridge School of Clinical Medicine, Cambridge, UK; 2 Department of Health Sciences, Faculty of Applied Health Sciences, Brock University, St Catharines, ON Canada; 3 MRC Epidemiology Unit, University of Cambridge, School of Clinical Medicine, Cambridge, UK

**Keywords:** Physical behaviour, Muscle mass, Sarcopenia, Longitudinal, Older adults, Physical activity, Sedentary, Muscle

## Abstract

**Background:**

The longitudinal associations between physical behaviours and lean muscle mass indices need to be better understood to aid healthy ageing intervention development.

**Methods:**

We assessed physical behaviours (total physical activity, moderate-to-vigorous physical activity (MVPA), light physical activity, total sedentary time and prolonged sedentary bout time) for 7 days using hip-worn accelerometers. We also assessed domain-specific physical behaviours (walking, cycling, gardening and housework time) with self-report questionnaires at baseline (2006–2011) and follow-up (2012–2016) in the European Prospective Investigation into Cancer (EPIC)-Norfolk study. We assessed body composition using dual-energy X-ray absorptiometry (DEXA) at follow-up in 1535 participants (≥ 60 years at baseline). From this, we derived appendicular lean muscle mass (ALM) indices (% relative ALM = (ALM/total body weight)*100), body mass index (BMI)-scaled ALM (ALM/BMI, kg/kg/m^2^) and height-scaled ALM (ALM/height^2^, kg/m^2^)). We evaluated the prospective associations of both baseline and change in physical behaviours with follow-up muscle mass indices using multivariable linear regression.

**Results:**

Over 5.5 years (SD 14.8) follow-up, higher baseline accelerometer-measured physical activity and lower sedentary time were associated with higher subsequent relative ALM and BMI-scaled ALM, but not height-scaled ALM (e.g. 0.02% higher subsequent relative ALM per minute/day of baseline MVPA for men). Greater increases in physical activity and greater declines in sedentary time variables were associated with higher subsequent relative ALM and BMI-scaled ALM, but not height-scaled ALM (e.g. 0.001 kg/kg/m^2^ subsequent BMI-scaled ALM and 0.04% subsequent relative ALM per min/day/year increases in LPA over follow-up; 0.001 kg/kg/m^2^ subsequent BMI-scaled ALM and -0.03% subsequent relative ALM per min/day/year less of total sedentary time over follow-up).

Greater increases in women’s cycling and gardening over follow-up were associated with greater subsequent relative ALM (cycling 0.9% per hour/week/year; gardening 0.2% per hour/week/year) and BMI-scaled ALM (cycling 0.03 kg/kg/m^2^ per hour/week/year; gardening 0.004 kg/kg/m^2^ per hour/week/year).

**Conclusion:**

Physical behaviours across all intensities, and in women more specifically cycling and gardening, may help prevent age-related declines in muscle mass.

**Supplementary Information:**

The online version contains supplementary material available at 10.1186/s12966-023-01547-6.

## Introduction

Muscle mass decreases between 3 and 8% per decade from age 30 [1]. By the age of 75 years, this rate of decline is 0.7% per year in women and up to 1% per year in men [2]. Declining muscle mass leads to reduced muscle function, as well as increased risk of physical disabilities, depression, nursing home admission and premature mortality [3]. Maintaining lean muscle mass is therefore an important component of healthy ageing [4]. Concurrently, high levels of physical activity (both aerobic and muscle-strengthening) and low levels of sedentary time are associated with lower risk of falls, dementia, diabetes, cardiovascular and cancer morbidity and premature mortality [5–8]. Chronic health conditions (e.g. diabetes, malignancies) are also risk factors for developing sarcopenia [9, 10].

Sarcopenia is defined as a muscle disease characterised by low muscle strength due to low muscle quantity and quality [11]. As muscle mass and quality are technically difficult to measure, sarcopenia is usually identified by poor physical function [11]. However, it is important for muscle mass to be studied independently as a marker of sarcopenia for several reasons. Firstly, muscle mass is only weakly correlated with physical function in older adults [12–16]. This is unsurprising given that muscle quality and proprioception/coordination also play roles in physical function. Secondly, muscle mass has other important roles beyond physical function. For example, skeletal muscle serves an important metabolic role in homeostasis of circulating substrates, heat regulation and providing amino acids for bodily function (e.g. in wound healing, immune functions and gluconeogenesis) [17].

Despite the benefits of an active lifestyle, older adults are not meeting recommended physical activity levels [18], and we currently do not have effective interventions for the maintenance of muscle mass throughout the life course. [19–21]. Although increases in physical activity and reductions in sedentary time are recommended for the prevention of muscle mass loss, [22] questions remain regarding the relationships between these factors. Firstly, existing studies have tended to use non-reference standard muscle mass indices such as bioelectrical impedence analysis [23–27] or DEXA indices which do not take body size into account (e.g. absolute ALM) [28–31].

Secondly, previous epidemiological studies have examined only the cross-sectional relationship between physical activity and muscle mass [32]. There have been no longitudinal studies, despite the importance of such studies in providing evidence for the degree and direction of these relationships over time. There are no studies of this relationship which have included repeated measures of activity. This is important as it is not clear if change in physical behaviours are associated with muscle mass, independent of baseline activity levels.

Thirdly, these cross-sectional studies have largely relied on self-reported measures of activity [24, 25, 29, 33–40]. The main reason that subjective measures continue to dominate the literature is that they are cheap and quick to administer. Common problems to all questionnaires are their vulnerability to recall bias, self-report bias, and the potential for responses to be influenced by cultural norms and perceived social desirability [41]. Accelerometer-assessed physical behaviours avoid these limitations.

Fourth, while maintaining and increasing physical activity is currently recommended for preventing muscle loss, no specific recommendations are currently made with regards to limiting sedentary time to prevent sarcopenia. Although a few early cross-sectional studies suggest that there is an association between high sedentary time and low muscle mass [42], there is no longitudinal research on this topic. No studies have examined the associations between breaking up prolonged sedentary bouts and muscle mass indices.

Finally, it is unclear which domain-specific physical activities (e.g. gardening, cycling) are associated with preventing reductions in muscle loss, as previous work using self-reported physical activity has predominantly focussed on total activity [26, 46, 47]. Though subjective measures have their limitations as outlined above, they allow individuals to be asked about their time in context-specific behaviours through questionnaires. It is important for us to understand these associations, given that it could inform recommendations on which domain-specific activities may provide the most benefit for guidelines on preventing sarcopenia and maintaining muscle mass on.

To better inform public health advice and the content of interventions for the preservation of muscle mass, we used data from the European Prospective Investigation into Cancer (EPIC)-Norfolk cohort to investigate the longitudinal associations of accelerometer-assessed intensity-specific and self-reported domain-specific physical behaviours with dual-energy X-ray absorptiometry (DEXA)-measured muscle mass.

## Methods

We used data previously collected from the EPIC-Norfolk cohort study, a prospective cohort of over 25,000 adults living in the UK [43]. Participants were recruited from primary care practices in Norfolk, UK between 1993 and 1997. Participants were comparable to a national population sample from the Health Survey for England in terms of anthropometry, serum lipids, and blood pressure [44]. Of the five health-checks conducted in the EPIC-Norfolk study, we used data from health-checks 3 and 4, which we describe herein as baseline (2006–2011) and follow-up (2012–2016). Physical behaviours (physical activity and sedentary time) were assessed at the baseline and follow-up health-checks. Muscle mass was measured using DEXA at follow-up (n = 5,573). The baseline and follow-up health-checks were attended by 7,312 and 4,992 participants aged ≥ 60 years, respectively. Available accelerometers were randomly assigned to individuals prior to each baseline visit. A total of 3,727 individuals agreed to wear an accelerometer at baseline (51%). At the follow-up health-check, all 4,992 individuals were asked to wear an accelerometer and 4,801 agreed (96%). Those who refused to wear accelerometers at follow-up were socio-demographically similar to those who were included (data not shown). We restricted our analyses to participants who were aged ≥ 60 years at baseline.

### Accelerometry

Hip-mounted accelerometers were used to collect data on time spent in different activity intensities at baseline and follow-up. Participants were asked to wear accelerometers on their right hip for seven days except when bathing, swimming or sleeping. During baseline assessment, participants wore uniaxial accelerometers (Actigraph GT1M™, USA) and at follow-up they wore triaxial accelerometers (GT3X™, Actigraph, USA). We harmonised data from uniaxial and triaxial accelerometers using previously described methods [45, 46]. Activity was integrated into counts per 60-s epochs (counts per min, cpm) [47, 48]. Variables derived from accelerometry data were total physical activity, MVPA (moderate-to-vigorous activity), LPA (light physical activity), total sedentary time, and prolonged sedentary bout time (bouts ≥ 30 min). We calculated total physical activity volume by summing accelerometer counts and dividing by wear time (expressed in counts/minute). The remaining accelerometry variables are durations (expressed in minutes/day). The movement intensity cut-offs used to define these time-based behaviour estimates were < 100 cpm for sedentary time, 100–808 cpm for LPA, and ≥ 809 cpm for MVPA [45, 49–53].

Non-wear time was defined as continuous zero counts of ≥ 90 min [53]. Overnight wear was dealt with by overlaying self-report sleep timings at the epoch level for days with wear-time > 19 h and then excluding data accordingly. Participants with ≥ 4 days of valid wear-time (≥ 10 h of wear time each day) were included in this analysis. We calculated the rate of change of accelerometer-assessed variables as the difference between values at baseline and follow-up divided by the time between assessments (min/day/year).

### Self-reported activity

The domain-specific physical activity types included were walking, cycling, gardening and housework. These were assessed using a season-specific (summer/winter) self-completed questionnaire (“In a typical week during the past year, how many hours did you spend on each of the following activities?”). We calculated average walking, cycling and gardening time (hours/week) by summing the respective summer and winter variables and dividing by two. Rate of change in walking, cycling, gardening and housework time were calculated as the difference between values at baseline and follow-up divided by the time between assessments (hours/week/year).

### Anthropometry

We assessed height (m) using stadiometers and body mass (kg) using calibrated scales during clinical health checks, performed by trained staff following standard operating procedures. BMI was calculated as body mass divided by height squared.

### Body composition

Body composition was assessed with whole-body DEXA, specifically using GE iDEXA utilising enCORE software version 14 (GE Healthcare). Participants were scanned by trained operators using standard imaging and positioning protocols [54]. Before scanning, DEXA systems were calibrated according to the manufacturer’s guidelines using a spine phantom made of calcium hydroxyapatite, embedded in a Lucite block (GE-Lunar, Madison, WI). The enCORE software automatically demarcated the boundaries of body regions which were checked and adjusted if needed by trained operators.

We derived the three most commonly used indices for lean muscle mass [55]. These included relative appendicular lean muscle mass (ALM) ((calculated by the sum of the lean tissue in the arms and legs/total body weight)*100), (units %)), BMI-scaled ALM (calculated by the sum of the lean tissue in the arms and legs divided by BMI, units kg/kg/m^2^) and height-scaled ALM (calculated by ALM divided by height squared, units kg/m^2^). All three indices were used in the analyses given that each index provides unique information and shows different patterns of change with age and sex [55].

### Covariates

Baseline sociodemographic factors included age, sex, smoking status (never, former, current), and occupational classification (*Registrar-General's Social Classification which has five categories;* I professional, II managerial/technical occupations III skilled occupations, IV partly skilled occupations and V unskilled occupations). We also assessed job status (job vs no job), educational status (completed educational qualification at aged 16 (English qualification is O level) or lower vs completed further education qualification at age 16–18 (English qualification is A level)), chronic disease status (history of either myocardial infarction, stroke, cancer or diabetes mellitus), and household financial circumstances (“in general, do you or your family have more money than you need, just enough or not enough?” Yes or No). All these were assessed via self-completed questionnaire.

### Statistical analysis

Descriptive statistics were calculated for all socio-demographic, physical activity and DEXA measures. We examined differences in socio-demographic characteristics of those participants that were included and those who were excluded from our main analyses.

For our primary association analyses, we estimated the associations between 1) baseline activity and follow-up muscle mass indices, and 2) change in activity from baseline to follow-up and follow-up muscle mass variables using multivariable linear regression. In these analyses, we examined accelerometer-assessed activity (time in total physical activity, MVPA, LPA, total sedentary time and prolonged sedentary bouts) and domain-specific self-report activity (time spent walking, cycling, gardening and housework).

We examined each of these associations using three differently adjusted models. Given that season, age and sex have repeatedly been found to be associated with physical activity, these factors are potential confounders [56–58]. Model 1 was adjusted for accelerometer wear time, season of each assessment [57, 59], time difference between baseline and follow-up, age and sex. Other biologically plausible confounders were fitted in to Model 2 (baseline job status, smoking status, chronic disease status, occupational class, and household financial circumstances). For the change in activity analyses, adjustment was also made for the baseline activity variable in question across all models.

We conducted sensitivity analyses to determine if the valid day inclusion criteria (≥ 5 vs ≥ 4 days of valid data) or the movement intensity cut-points (i.e. 809 vs ≥ 2,020 cpm for MVPA; 100–808 vs 100–2,019 cpm for LPA) influenced results. All analyses were conducted in STATA 15.0 (StataCorp, TX, USA) using complete case analyses.

## Results

There were 1,612 participants adults aged ≥ 60 who had muscle mass and activity measurements at both health-checks making them eligible for inclusion. Of these, 44 individuals were excluded due to having < 4 valid days of accelerometry data (n = 22 at baseline, *n* = 22 at follow-up) and 33 individuals were excluded for having missing covariate data, leaving a total of 1,535 participants (95%). Participants had an average age of 68.7 years at baseline (SD 6.0) and 55.5% were women (Table 1). Included participants were socio-demographically similar to those excluded (Supplementary Table 1). Mean time between baseline and follow-up was 5.5 years (SD 1.9). On average, MVPA decreased by 3.8 min/day/year (SD 8.5) for men and 3.6 min/day/year for women (SD 8.7) from baseline to follow-up. Total sedentary time increased by an average of 5.5 min/day/year (SD 16.5) for men and 6.4 min/day/year (SD 14.8) for women from baseline to follow-up (Table 2, Supplementary Table 2). Rates of participation in baseline and follow-up domain-specific activities were adequate for assessment of change analyses (e.g. baseline cycling 29.2% men and 20.2% women; follow-up, 25.2% men and 18.0% women) (Supplementary Table 3).

**Table 1:** Baseline demographic characteristics

CATEGORY	Subcategory	Frequency (%)	
		**Male**	**Female**
**Ethnicity**	White	681 (99.7)	852 (100)
	Other	2 (0.3)	0 (0)
**Occupational Classification**	Professional	71 (10.4)	66 (7.8)
	Manager	295 (43.2)	371 (43.5)
	Skilled non-manual	71 (10.4)	139 (16.3)
	Skilled manual	165 (24.2)	164 (19.3)
	Semi-skilled	69 (10.1)	94 (11.0)
	Non-skilled	12 (1.8)	18 (2.1)
**Further Education level**	O-level or lower	342 (50.1)	376 (44.1)
	A-level or higher	341 (49.9)	476 (55.9)
**History of Chronic Disease**	No	567 (83.0)	730 (85.7)
	Yes	116 (17.0)	122 (14.3)
**Smoking Status**	Current	12 (1.8)	25 (2.9)
	Former	395 (57.8)	301 (35.3)
	Never	276 (40.4)	526 (61.7)
**Employed**	No	488 (71.5)	690 (81.0)
	Yes	195 (28.6)	162 (19.0)
**Body Mass Index (kg/m**^**2**^**)**	< 25	203 (29.7)	362 (42.4)
	25- < 30	349 (51.1)	349 (41.0)
	30- < 35	108 (15.8)	106 (12.4)
	≥ 35	23 (3.4)	35 (4.1)

This table shows the percentage spread across categories of demographic and clinical *characteristics* for those included (*n* = 1,535). Participants had an average age of 68.7 years at baseline (SD 6.0) and 74.2 years at follow-up (SD 6.1), with 55.5% being female (Men *n* = 683, women *n* = 852). Further education level categories include O level or lower (UK national qualification to age 16) vs A level or higher (UK national qualification over age 16). Baseline characteristics here were undertaken in 2006–2011

**Table 2:** Exposure and outcome characteristics

Exposure or Outcome	Baseline Mean (SD)		Follow-up Mean (SD)		Mean Annual Change (SD)	
	**Male**	**Female**	**Male**	**Female**	**Male**	**Female**
**TPA (cpm)**	279 (124.6)	269.9 (109.1)	229.2 (124.0)	224.8 (104.3)	-9.0 (22.4)	-8.9 (20.2)
**Total sedentary time (min/day)**	571 (81.1)	532.6 (77.4)	595.3 (75.3)	564.1 (78.6)	5.5 (16.5)	6.4 (14.8)
**Prolonged sedentary bout (min/day)**	211.3 (92.7)	167.4 (82.6)	261.8 (106.4)	213.4 (96.6)	9.3 (19.8)	8.9 (17.0)
**LPA **^**100–809 cpm**^** (min/day)**	216.7 (53.3)	244.8 (51.9)	195.3 (55.3)	225.5 (56.4)	-4.0 (11.4)	-4.0 (12.)
**MVPA **^**809 cpm**^** (min/day)**	89.5 (48.3)	83.8 (45.0)	69.2 (43.9)	65.9 (40.9)	-3.8(8.5)	-3.6 (8.7)
**Walking time (hours/week)**	9.1 (7.9)	9.0 (8.8)	10.0 (10.4)	9.0 (8.7)	-0.2 (2.7)	-0.04 (2.4)
**Cycling time (hours/week)**	1.0 (3.0)	0.6 (2.1)	1.0 (4.4)	0.5 (1.7)	-0.004 (0.8)	-0.04 (0.5)
**Gardening time (hours/week)**	6.3 (7.1)	4.5 (5.6)	6.6 (7.2)	4.8 (6.1)	0.03 (1.7)	0.003 (1.8)
**Housework time (hours/week)**	5.2 (7.9)	16.5 (12.4)	6.8 (8.6)	16.3 (12.9)	0.3 (2.2)	-0.01 (3.5)
**Total lean muscle mass (kg)**	-	-	24.0 (3.2)	16.7 (2.6)	-	-
**Total lean appendicular muscle mass (kg)**	-	-	52.8 (6.1)	38.5 (4.8)	-	-
**Height-scaled ALM (kg/m**^**2**^**)**	-	-	8.0 (0.9)	6.5 (0.9)	-	-
BMI-scaled **ALM (kg/kg/m**^**2**^**)**	-	-	0.9 (0.1)	0.6 (0.8)	-	-
**Relative ALM (%)**	-	-	29.7 (2.8)	24.8 (2.5)	-	-

This table shows the mean values of activity measures and muscle mass measures at baseline and follow-up. Baseline measurements were undertaken between 2006–2011, and follow-up between 2012–2016. *TPA* Total physical activity, *MVPA* Moderate-to-vigorous activity, *LPA* Light physical activity, *SD* Standard deviation, *ALM* Appendicular lean muscle mass, *BMI* Body mass index. A dash denotes that the measure was not taken in the assessment

### Association of baseline physical behaviours with follow-up muscle mass variables (Supplementary Table 4)

#### *Accelerometer-assessed physical behaviours* (Fig. 1A)

**Fig. 1:**
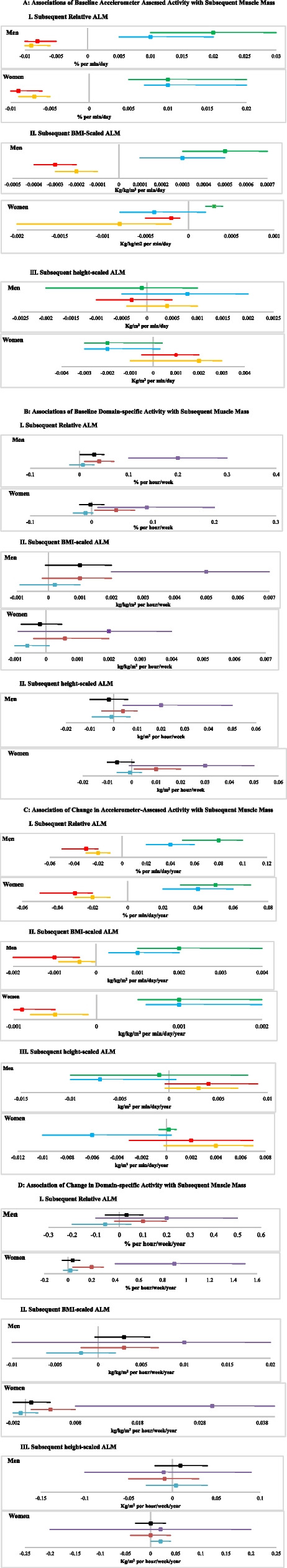
For all **A** and **C**, MVPA is in green, LPA is in blue, total ST is in red and Prolonged ST bouts is in orange. For **B** and **D**, walking time is in black, cycling time is in purple, gardening time is in red and housework time is in blue. Beta is indicated by central square, 95% CI is indicated by the line. Baseline measures were taken between 2006–2011 and follow-up measures were taken between 2012–2016. Change in variables was from baseline to follow-up. In **A** and **B**, results are from model 3 adjusted for season and wear time at baseline, baseline age, sex, job status, smoking status, occupational class, household financial status, and chronic disease status. In **C** and **D**, season and wear time at baseline and follow-up, age, sex, job status, smoking status, occupational class, retirement status, household financial status, chronic disease status, and baseline activity. ALM appendicular lean muscle mass, BMI = body mass index, h = height.

Higher baseline physical activity and lower sedentary time were associated with higher subsequent muscle mass indices (relative ALM and BMI-scaled ALM but not height-scaled ALM for both men and women). Every 100 cpm of baseline total physical activity was associated with a 0.7% higher relative ALM and 0.02 kg/kg/m^2^ higher BMI-scaled ALM for men and women. Every minute/day of baseline physical activity was associated with higher subsequent relative ALM (MVPA men 0.02%, women 0.01%; LPA men and women 0.01%) and BMI-scaled ALM (MVPA men 0.0005 kg/kg/m^2^, women 0.0003 kg/kg/m^2^; LPA men 0.0003 kg/kg/m^2^, women no association). Every minute/day of baseline sedentary time was associated with lower subsequent relative ALM (total sedentary time men -0.008%, women -0.009%; prolonged sedentary bout time men -0.009%, women -0.007%) and BMI-scaled ALM (total sedentary time men -0.0003 kg/kg/m^2^, women -0.0001 kg/kg/m^2^; prolonged sedentary bout time bouts men -0.0002 kg/kg/m^2^, women -0.0008 kg/kg/m^2^).

#### Domain-specific physical activity (Fig. 1B)

Higher baseline walking time was associated with higher subsequent relative ALM for men only (0.03% per hour/week walking). Each hour/week of baseline cycling time was associated with greater relative ALM (men 0.2%, women 0.09%), BMI-scaled ALM (men 0.005 kg/kg/m^2^, women no association) and height-scaled ALM (men 0.02 kg/m^2^, women no association). Each hour/week of baseline gardening time was associated with greater relative ALM (men and women 0.04%). Baseline housework was not associated with any follow-up muscle mass indices.

### Association of change in physical behaviour with muscle mass at follow-up (Supplementary Table 5)

#### *Accelerometer-assessed physical behaviours* (Fig. 1C)

Greater increases in physical activity and greater reductions in sedentary time over time were associated with higher subsequent relative ALM and BMI-scaled ALM, but not height-scaled ALM. Greater increases in total physical activity were associated with higher subsequent relative ALM (men: 3% per 100 cpm/year, women 2% per 100 cpm/year) and BMI-scaled ALM (men 0.09 kg/kg/m^2^ per 100 cpm/year, women 0.06 kg/kg/m^2^ per 100 cpm/year). Greater increases in physical activity were associated with higher subsequent relative ALM (MVPA men 0.08% per min/day/year, women 0.05% per min/day/year; LPA men and women 0.04% per min/day/year) and BMI-scaled ALM (MVPA men 0.002 kg/kg/m^2^ per min/day/year, women 0.001 kg/kg/m^2^ per min/day/year; LPA men and women 0.001 kg/kg/m^2^ per min/day/year).

Greater increases in sedentary time were associated with lower subsequent relative ALM (total sedentary time men and women -0.03% per min/day/year; prolonged sedentary bout time men and women -0.02% per min/day/year) and BMI-scaled ALM (total sedentary time men -0.001 kg/kg/m^2^, women -0.0009 kg/kg/m^2^; prolonged sedentary bout time men -0.0004 kg/kg/m^2^ per min/day/year, women -0.0005 kg/kg/m^2^ per min/day/year).

#### *Domain-specific activity* (Fig. 1D)

For men, there were no associations between change in duration of any domain-specific activities and subsequent muscle mass indices. For women, greater increases in cycling and gardening were associated with greater subsequent relative ALM (cycling 0.9% per hour/week/year; gardening 0.2% per hour/week/year), BMI-scaled ALM (cycling 0.03 kg/kg/m^2^ per hour/week/year; gardening 0.004 kg/kg/m^2^ per hour/week/year). Greater increases in housework were associated with greater height-scaled ALM (housework 0.02 kg/m^2^ per hour/week/year).

### Sensitivity analyses

The results were similar when different cut-points for MVPA and LPA were utilised, and also when using the stricter inclusion criteria of ≥ 5 days of valid accelerometer wear-time versus the criteria of ≥ 4 days of valid wear-time (Supplementary Tables 4 and 5).

## Discussion

We found that individuals with higher baseline physical activity (LPA and MVPA) and lower sedentary time levels (total time and prolonged sedentary bout time) had higher subsequent muscle mass indices (relative ALM and BMI-scaled ALM, but not height-scaled ALM) an average of 5.5 years later. Men and women who cycled and gardened more at baseline had higher levels of at least muscle mass index at follow-up, and men who walked more at baseline had higher subsequent relative ALM. Further, those who had greater increases in their MVPA and LPA time, or greater declines in their total sedentary time or prolonged sedentary bout time over follow-up, had better subsequent muscle mass indices. In women, those who had greater increases in their cycling, gardening or housework time over follow-up had higher subsequent levels of at least one muscle mass index.

To our knowledge, no previous studies have examined the association between baseline accelerometer-assessed physical behaviours with follow-up muscle mass assessed by DEXA in adults of any age, or change in accelerometer-assessed physical behaviours with follow-up muscle mass indices. Studies examining the relationships between physical behaviours and muscle mass have been exclusively cross-sectional [60]. Of these cross-sectional studies, they have almost exclusively used self-reported measures of total physical activity [24, 25, 29, 33–40] which are prone to recall and self-report bias. Three studies have produced mixed results concerning whether cross-sectional associations exist between accelerometer-assessed activity and muscle mass indices. Westbury and colleagues found no association between MVPA and height-scaled ALM among 131 UK older adults [61], whereas Foong and colleagues found that higher LPA and MVPA were associated with higher relative ALM in 636 Australian older adults. Reid and colleagues found an association between higher total sedentary time and lower total lean mass percentage, but not BMI-scaled ALM or height-scaled ALM, in a group of older Australians (*n* = 123) [62].

Our study is in agreement with these associations, but it is the first to demonstrate positive prospective associations between MVPA and LPA and muscle mass indices, and negative associations between sedentary variables and muscle mass indices using objective standard reference measures in older adults. Where cross-sectional studies cannot rule out reverse causality, our data strengthens the likelihood of direction of the associations between physical behaviours and muscle mass indices found here.

We are additionally the first to report on the associations of change in physical behaviours with subsequent muscle mass indices. Where cross-sectional work supports the notion that physical behavioural interventions leading to achievement of an absolute time in physical activity (e.g. 30 min of MVPA/day) may be beneficial to muscle mass profiles, our work suggests that a change in time in activity (e.g. an increase of 10 min of MVPA /day) might be beneficial independent of the baseline activity level.

We know of no existing studies examining the prospective association of domain-specific activity types with muscle mass indices. Even the cross-sectional data on this topic are scarce, with only two studies looking at leisure-time versus work-time physical activity [26, 63], and one examining walking time [64]. The authors of this latter study [64] found that walking time was negatively associated with sarcopenia (defined as ALM/height^2^ < 2SD below the sex-specific normal mean for the younger reference group, OR = 0.49, 95% CI 0.29–0.83). In contrast, we found no associations between walking and height-scaled ALM, though we did find an association between baseline walking and subsequent relative ALM in men.

We are the first to report that men and women who cycle and garden more have better subsequent muscle mass indices values. Further, in women we are the first to report that higher increases or lower declines in cycling, gardening or housework time were associated with higher subsequent levels of at least one muscle mass index. It is plausible that baseline gardening and cycling time are predictors of future muscle mass, given the strength component in both activities (engaging two of the largest muscle groups in the body, the gluteals and quadriceps, in peddling and digging). Understanding which activities may be most useful in maintaining muscle mass indices and preventing sarcopenia is important for informing what activities should be targeted in future interventions. Given that that cycling and gardening are neglected targets in previous RCTs aiming to prevent sarcopenia, this highlights a potential area for future work [65].

Relative ALM, BMI-scaled ALM, and height-scaled ALM are the commonest operational indices used in lean muscle mass research to assess sarcopenia. Each of these indices have been found to have different patterns of change with ageing and with sex [55]. Muscle mass is fundamentally correlated with body size (i.e. individuals with a larger body habitus, by either height or weight, have larger absolute muscle mass) [66]. This is intuitive given that taller individuals would be expected to have longer limbs and therefore greater appendicular muscle attached to them, and heavier individuals would have greater appendicular muscle from lifting their heavier limbs than their lighter counterparts. Our findings suggest that physical activity has an effect on ALM indices which take into account weight or BMI, but not those only accounting for height. Studies have previously found that BMI-scaled ALM is more closely related to cardiometabolic risk factors than height-scaled ALM [67], suggesting the former may be a better risk marker. It is important to note that reductions in relative ALM or BMI-scaled ALM may result from a reduction in absolute ALM or an increase in total body weight (and hence BMI) for another reason (e.g. an increase in body fat). Regardless, both result in proportionally less appendicular lean muscle to move each kg of total weight, which will functionally mean it is potentially harder to move.

We found that reductions in sedentary time of the magnitude previously achieved in RCTs (1 h/day/year) would lead to a 1.8%/year higher relative ALM (both men and women) and 0.06 kg/kg/m^2^ higher BMI-scaled ALM for men and 0.05 kg/kg/m^2^ for women [68, 69]. We also found that improvements in MVPA of the magnitude seen in RCTs (10 min/day/year) would lead to a 0.8%/year increase in subsequent relative ALM for men and 0.5%/year for women and 0.02 kg/kg/m^2^ improvement in BMI-scaled ALM for men and 0.01 kg/kg/m^2^ for women [69, 70]. To give some context to these magnitudes of change, we observed age-related declines in muscle mass indices in this cohort of 0.04% relative ALM per year of older age and 0.004 kg/kg/m^2^ of BMI-scaled ALM per year of older age. Therefore, it is conceivable that if sustained positive change in physical behaviours was achieved in this population the potential improvements in muscle mass indices could offset the age-associated declines we observed in this cohort. In total, this suggests that further investigation of interventions that aim to improve physical behaviour profiles is warranted as there is potential for improvements in muscle mass indices which could reduce sarcopenia.

It is important to note that that improvements in MVPA were beneficial to muscle mass indices independent of baseline MVPA levels. Here, we report that a 10 min/day/year increase in MVPA led to a 0.8%/year higher subsequent relative ALM in men and a 0.5%/year higher subsequent relative ALM in women respectively. To compare this with the benefit of greater baseline MPVA, for each 10 min of baseline MVPA, individuals had 0.2% and 0.1% greater subsequent relative ALM, for men and women, respectively. Therefore, there seems to be a role for promoting both change in activity in addition to achieving optimal absolute activity levels.

Our work also suggests that promoting LPA and reducing prolonged sedentary bout time, potentially easier targets than the promotion of MVPA, could also lead to improvement in muscle mass indices. Though there is insufficient evidence of achievable improvements in LPA from the literature, if we assume that increases of 10 min/day/year are possible (as it is for MVPA), we found that improvements in LPA of 10 min/day/year would lead to a 0.4%/year improvement in subsequent relative ALM and 0.01 kg/kg/m^2^ improvement in BMI-scaled ALM. Although this is a lower benefit than for the same amount of MVPA change, it may be easier to encourage individuals to increase LPA rather than MVPA. If we assume that 1 h/day reduction in prolonged sedentary bout time is possible (as it has been in RCTs for total sedentary time), we found that reductions of this magnitude led to a 1.2%/year improvement in subsequent relative ALM and 0.02 kg/kg/m^2^ improvement in BMI-scaled ALM. Again, although this is a lower benefit per minute than for total sedentary time change, it may be easier to encourage individuals to reduce prolonged sedentary bout time through taking breaks, than reducing total sedentary time. Breaking up prolonged sedentary bouts is now recommended in the Canadian physical activity guidelines [71]. Our findings add improvements in muscle mass indices to the other known benefits of breaking up prolonged sitting, such as improved glycaemic control [72].

The UK activity guidelines give practical examples of muscle strengthening such as carrying heavy shopping bags, yoga, lifting weights, doing resistant band-work, body weight exercises or heavy gardening [73]. Our findings support this, and additionally indicate that walking, cycling and housework may contribute to maintaining muscle mass into older age.

### Strengths and limitations

The strengths of this work include utilising a large well-characterised longitudinal population-based cohort study which increases reliability and power. Particularly, the use of repeat accelerometer-assessed physical activity and sedentary time rather than only self-report measures, and reference standard DEXA measurement of muscle mass indices which take into account body size is a great strength.

This work also has some limitations. Hip-mounted accelerometers, which cannot be worn at all times, will provide an incomplete record of physical activity, although this is still superior in accuracy to self-report measures. A single accelerometer on the hip is not able to collect relevant information on upper body movement, standing still vs sitting down, cycling, and do not record water-based activity such as swimming which is popular among some older participants. We minimised non-wear misclassification (i.e. not wearing the accelerometer versus being still) using standard protocols (algorithm with a threshold of ≥ 90 min) [74]. Common to other cohort studies, EPIC-Norfolk participants were slightly healthier than the general population [44] at the first health-check (1993–1997), and third health-check (2004–2011) as indicated by blood pressure and cholesterol levels [43]. Third health-check attendees were more likely to be younger, have lower BMI, blood pressure and cholesterol and more likely to be educated to at least to aged 16 than first health-check attendees [43]. Further, the EPIC-Norfolk cohort is predominantly white British cohort limiting the generalisability of this data to other ethnicities. Overall, these selection pressures could limit generalisability of results. In spite of these limitations, this dataset still represents a diverse population with a wide socioeconomic distribution.

## Conclusions

Physical activity declines and sedentary behaviours increase over time in old age. Greater increases in physical activity and greater declines in sedentary time, were associated with higher subsequent lean muscle mass relative to overall body size. Taken together, this supports the case for development of effective physical behaviour interventions (at all intensities and in the domains of walking, cycling and gardening, in particular for women) given that sustained changes in these behaviours may help prevent age-related declines in muscle mass. Future researchers may wish to focus more on LPA, sedentary time and prolonged sedentary bouted time, which have previously been neglected but may be easier targets for change. Lean muscle mass outcomes, such as those used in our study, should be considered in the design of future trials and cost-effectiveness analyses.

### Supplementary Information

**Additional file 1:** **Table S1.** Participant characteristics for those included versus excluded.

**Additional file 2:** **Table S2.** Exposure characteristics for alternative cut-points of LPA and MVPA.

**Additional file 3:** **Table S3.** Participation Rates of domain-specific activities.

**Additional file 4:** **Table S4.** Association of baseline physical activity and sedentary time with follow-up muscle mass, Men n=683, Women n=852.

**Additional file 5:** **Table S5.** Association of change in physical activity and sedentary time with follow-up muscle mass.

## Data Availability

Data from the EPIC-Norfolk study must be requested directly from their data request team by completing a data request form.

## Abbreviations

MVPA: Moderate-to-vigorous physical activity
DEXA: Dual-energy X-ray absorptiometry
ALM: Appendicular lean muscle mass
BMI: Body mass index
LPA: Light physical activity
EPIC: European Prospective Investigation into cancer

## References

[1] Volpi E, Nazemi R, Fujita S. Muscle tissue changes with aging. Curr Opin Clin Nutr Metab Care. 2004;7(4):405. Available from: https://journals.lww.com/co-clinicalnutrition/abstract/2004/07000/muscle_tissue_changes_with_aging.9.aspx. 10.1097/01.mco.0000134362.76653.b2 PMC2804956 15192443

[2] Mitchell WK, Williams J, Atherton P, Larvin M, Lund J, Narici M, et al. Sarcopenia, dynapenia, and the impact of advancing age on human skeletal muscle size and strength; a quantitative review. 2012 [cited 2022 May 9]; Available from: www.frontiersin.org 10.3389/fphys.2012.00260 PMC3429036 22934016

[3] Beaudart C, Zaaria M, Pasleau F, Reginster JY, Bruyère O. Health Outcomes of Sarcopenia: A Systematic Review and Meta-Analysis. PLoS One. 2017;12(1):e0169548. https://journals.plos.org/plosone/article?id=. 10.1371/journal.pone.0169548. Available from: Cited 2021 July 21. 10.1371/journal.pone.0169548 PMC5240970 28095426

[4] McPhee JS, French DP, Jackson D, Nazroo J, Pendleton N, Degens H. Physical activity in older age: perspectives for healthy ageing and frailty. Biogerontology. 2016;17(3):567. 10.1007/S10522-016-9641-0. 10.1007/s10522-016-9641-0 PMC4889622 26936444

[5] Kouloutbani K, Karteroliotis K, Politis A. The effect of physical activity on dementia [Internet]. Vol. 30, Psychiatrike = Psychiatriki. Psychiatriki; 2019 [cited 2020 Jul 1]. p. 142–55. Available from: https://pubmed.ncbi.nlm.nih.gov/31425142/10.22365/jpsych.2019.302.142 31425142

[6] Thibaud M, Bloch F, Tournoux-Facon C, Brèque C, Rigaud AS, Dugué B, et al. Impact of physical activity and sedentary behaviour on fall risks in older people: a systematic review and meta-analysis of observational studies. European Review of Aging and Physical Activity. 2012;9(1):5–15. http://link.springer.com/10.1007/s11556-011-0081-1 Available from: cited 2020 Jun 30

[7] Krebs S, Berling-Ernst A, Halle M. Physical Activity and Cancer. Sportverletzung-Sportschaden. 2018;32(2):143–7. https://pubmed.ncbi.nlm.nih.gov/28859204/ Available from: Cited 2020 Jul 1 10.1055/s-0043-115187 28859204

[8] Ekelund U, Brown WJ, Steene-Johannessen J, Fagerland MW, Owen N, Powell KE, et al. Do the associations of sedentary behaviour with cardiovascular disease mortality and cancer mortality differ by physical activity level? A systematic review and harmonised meta-analysis of data from 850 060 participants [Internet]. Vol. 53, British Journal of Sports Medicine. Br J Sports Med; 2019. p. 886–94. https://pubmed.ncbi.nlm.nih.gov/29991570/ Available from: Cited 2020 Jul 1 10.1136/bjsports-2017-098963 29991570

[9] Therakomen, V., Petchlorlian, A., & Lakananurak, N. (2020). Prevalence and risk factors of primary sarcopenia in community-dwelling outpatient elderly: a cross-sectional study. Sci Rep. 10(1). 10.1038/S41598-020-75250-Y. 10.1038/s41598-020-75250-y PMC7658996 33177536

[10] Marzetti E, Calvani R, Tosato M, Cesari M, di Bari M, Cherubini A (2017). Sarcopenia: an overview. Aging Clin Exp Res.

[11] Cruz-Jentoft AJ, Bahat G, Bauer J, Boirie Y, Bruyère O, Cederholm T, Cooper C, Landi F, Rolland Y, Sayer AA, Schneider SM, Sieber CC, Topinkova E, Vandewoude M, Visser M, Zamboni M, Bautmans I, Baeyens J-P, Cesari M, Schols J. Sarcopenia: revised European consensus on definition and diagnosis. Age and Ageing. 2019;48(1):16–31. 10.1093/ageing/afy169. 10.1093/ageing/afy169 PMC6322506 30312372

[12] Kim KE, Jang S nang, Lim S, Park YJ, Paik NJ, Kim KW, et al. Relationship between muscle mass and physical performance: is it the same in older adults with weak muscle strength? Age Ageing. 2012;41(6):799–803. https://pubmed.ncbi.nlm.nih.gov/22910301/ Available from: Cited 2022 Aug 17 10.1093/ageing/afs115 22910301

[13] Chen L, Nelson DR, Zhao Y, Cui Z, Johnston JA. Relationship between muscle mass and muscle strength, and the impact of comorbidities: A population-based, Cross-sectional study of older adults in the United States. BMC Geriatr. 2013;13(1):1–8. https://bmcgeriatr.biomedcentral.com/articles/10.1186/1471-2318-13-74 Available from: Cited 2022 Aug 17 10.1186/1471-2318-13-74 PMC3765109 23865675

[14] Huang SW, Hsieh FC, Lin LF, de Liao C, Ku JW, Hsiao DJ (2018). Correlation between Body Composition and Physical Performance in Aged People. Int J Gerontol.

[15] Iwase H, Murata S, Nakano H, Shiraiwa K, Abiko T, Goda A, Nonaka K, Anami K, Horie J. Relationship between age-related changes in skeletal muscle mass and physical Function: a cross-sectional study of an elderly Japanese population. Cureus. 2022;14(4):e24260. 10.7759/cureus.24260. 10.7759/cureus.24260 PMC9123344 35607534

[16] Williams GR, Deal AM, Muss HB, Weinberg MS, Sanoff HK, Nyrop KA, et al. Skeletal muscle measures and physical function in older adults with cancer: sarcopenia or myopenia? Oncotarget. 2017;8(20):33658. /pmc/articles/PMC5464899/ Available from: Cited 2022 Aug 17 10.18632/oncotarget.16866 PMC5464899 28431396

[17] Research I of M (US) C on MN. Regulation of Muscle Mass and Function: Effects of Aging and Hormones. 1999; https://www.ncbi.nlm.nih.gov/books/NBK224631/ Available from: Cited 2022 Aug 17

[18] Statistics on Physical activity - NHS Digital. https://digital.nhs.uk/data-and-information/publications/statistical/statistics-on-obesity-physical-activity-and-diet/statistics-on-obesity-physical-activity-and-diet-england-2019/part-5-adult-physical-activity Available from: Cited 2020 Jul 1

[19] Baxter S, Blank L, Johnson M, Everson-Hock E, Woods HB, Goyder E, et al. Interventions to promote or maintain physical activity during and after the transition to retirement: an evidence synthesis. Public Health Research [Internet]. 2016;4(4):1–354. https://www.journalslibrary.nihr.ac.uk/phr/phr04040/ Available from: Cited 2016 Nov 22 27148615

[20] Gardner B, Smith L, Lorencatto F, Hamer M, Biddle SJ. How to reduce sitting time? A review of behaviour change strategies used in sedentary behaviour reduction interventions among adults. Health Psychol Rev [Internet]. 2016;10(1):89–112. http://www.tandfonline.com/doi/full/10.1080/17437199.2015.1082146 Available from: Cited 2016 Jul 17 10.1080/17437199.2015.1082146 PMC4743603 26315814

[21] Yoshimura Y, Wakabayashi H, Yamada M, Kim H, Harada A, Arai H. Interventions for Treating Sarcopenia: A Systematic Review and Meta-Analysis of Randomized Controlled Studies. J Am Med Dir Assoc [Internet]. 2017;18(6):553.e1–553.e16. http://www.jamda.com/article/S1525861017301901/fulltext Available from: Cited 2022 Jan 26 10.1016/j.jamda.2017.03.019 28549707

[22] Denison HJ, Cooper C, Sayer AA, Robinson SM (2015). Prevention and optimal management of sarcopenia: a review of combined exercise and nutrition interventions to improve muscle outcomes in older people. Clin Interv Aging.

[23] Shephard RJ, Park H, Park S, Aoyagi Y. Objectively measured physical activity and progressive loss of lean tissue in older Japanese adults: longitudinal data from the Nakanojo study. J Am Geriatr Soc. 2013;61(11):1887–93. https://pubmed.ncbi.nlm.nih.gov/24219190/ Available from: Cited 2022 Jan 17 10.1111/jgs.12505 24219190

[24] Therakomen V, Petchlorlian A, Lakananurak N. Prevalence and risk factors of primary sarcopenia in community-dwelling outpatient elderly: a cross-sectional study. Sci Rep. 2020;10(1). https://pubmed.ncbi.nlm.nih.gov/33177536/ Available from: cited 2022 Jan 17 10.1038/s41598-020-75250-y PMC7658996 33177536

[25] Ko YC, Chie WC, Wu TY, Ho CY, Yu WR. A cross-sectional study about the relationship between physical activity and sarcopenia in Taiwanese older adults. Sci Rep. 2021;11(1). https://pubmed.ncbi.nlm.nih.gov/34075104/ Available from: cited 2022 Jan 17 10.1038/s41598-021-90869-1 PMC8169879 34075104

[26] Zogg S, Dürr S, Maier S, Tomatis L, Uehli K, Miedinger D, et al. Relationship between domain-specific physical activity and different body composition measures in a working population. J Occup Environ Med [Internet]. 2014;56(10):1074–81. https://pubmed.ncbi.nlm.nih.gov/25285830/ Available from: Cited 2022 Jan 17 10.1097/JOM.0000000000000224 25285830

[27] Gába A, Pelclová J, Štefelová N, Přidalová M, Zając-Gawlak I, Tlučáková L, et al. Prospective study on sedentary behaviour patterns and changes in body composition parameters in older women: A compositional and isotemporal substitution analysis. Clin Nutr. 2021;40(4):2301–7. https://pubmed.ncbi.nlm.nih.gov/33109393/ Available from: cited 2022 Jan 17 10.1016/j.clnu.2020.10.020 33109393

[28] Alkahtani S, Aljuhani O, Alhussain M, Habib SS. Association between physical activity patterns and sarcopenia in Arab men. J Int Med Res. 2020;48(4):030006052091869. 10.1177/0300060520918694. 10.1177/0300060520918694 PMC7175057 32314627

[29] Morris MS, Jacques PF. Total protein, animal protein and physical activity in relation to muscle mass in middle-aged and older Americans. Br J Nutr. 2013;109(7):1294–303. https://pubmed.ncbi.nlm.nih.gov/22856586/ Available from: Cited 2022 Jan 17 10.1017/S0007114512003133 22856586

[30] Scott D, Johansson J, Gandham A, Ebeling PR, Nordstrom P, Nordstrom A. Associations of accelerometer-determined physical activity and sedentary behavior with sarcopenia and incident falls over 12 months in community-dwelling Swedish older adults. J Sport Health Sci. 2021;10(5):577–84. https://pubmed.ncbi.nlm.nih.gov/34088651/ Available from: Cited 2022 Jan 17 10.1016/j.jshs.2020.01.006 PMC8500807 34088651

[31] Savikangas T, Tirkkonen A, Alen M, Rantanen T, Fielding RA, Rantalainen T, et al. Associations of physical activity in detailed intensity ranges with body composition and physical function. a cross-sectional study among sedentary older adults. Eur Rev Aging Phys Act. 2020;17(1). https://pubmed.ncbi.nlm.nih.gov/31998411/ Available from: Cited 2022 Jan 17 10.1186/s11556-020-0237-y PMC6982388 31998411

[32] Steffl M, Bohannon RW, Sontakova L, Tufano JJ, Shiells K, Holmerova I. Relationship between sarcopenia and physical activity in older people: a systematic review and meta-analysis. Clin Interv Aging. 2017;12:835–45. https://pubmed.ncbi.nlm.nih.gov/28553092/ Available from: Cited 2022 Jan 17 10.2147/CIA.S132940 PMC5441519 28553092

[33] Hwang AC, Zhan YR, Lee WJ, Peng LN, Chen LY, Lin MH, et al. Higher Daily Physical Activities Continue to Preserve Muscle Strength After Mid-Life, But Not Muscle Mass After Age of 75. Medicine. 2016;95(22). https://pubmed.ncbi.nlm.nih.gov/27258519/ Available from: Cited 2022 Jan 17 10.1097/MD.0000000000003809 PMC4900727 27258519

[34] Huschtscha Z, Parr A, Porter J, Costa RJS. Sarcopenic characteristics of active older adults: a cross-sectional exploration. Sports Med Open. 2021;7(1):32. 10.1186/s40798-021-00323-9. 10.1186/s40798-021-00323-9 PMC8128944 33999277

[35] Trajanoska K, Schoufour JD, Darweesh SKL, Benz E, Medina-Gomez C, Alferink LJM, et al. Sarcopenia and Its Clinical Correlates in the General Population: The Rotterdam Study. J Bone Miner Res. 2018;33(7):1209–18. https://pubmed.ncbi.nlm.nih.gov/29502340/ Available from: cited 2022 Jan 17 10.1002/jbmr.3416 29502340

[36] Oh C, Jeon BH, Reid Storm SN, Jho S, No JK. The most effective factors to offset sarcopenia and obesity in the older Korean: Physical activity, vitamin D, and protein intake. Nutrition. 2017;33:169–73. https://pubmed.ncbi.nlm.nih.gov/27717662/ Available from: Cited 2022 Jan 17 10.1016/j.nut.2016.06.004 27717662

[37] Balogun S, Aitken D, Winzenberg T, Wills K, Scott D, Callisaya M, et al. Longitudinal Associations of Serum 25-hydroxyvitamin D, Physical Activity, and Knee Pain and Dysfunction with Muscle Loss in Community-dwelling Older Adults. J Gerontol A Biol Sci Med Sci. 2018;73(4):526–31. https://pubmed.ncbi.nlm.nih.gov/28958061/ Available from: Cited 2022 Jan 17 10.1093/gerona/glx157 28958061

[38] Baumgartner RN, Waters DL, Gallagher D, Morley JE, Garry PJ. Predictors of skeletal muscle mass in elderly men and women. Mech Ageing Dev. 1999;107(2):123–36. https://pubmed.ncbi.nlm.nih.gov/10220041/ Available from: Cited 2022 Jan 17 10.1016/s0047-6374(98)00130-4 10220041

[39] Eibich P, Buchmann N, Kroh M, Wagner GG, Steinhagen-Thiessen E, Demuth I, et al. Exercise at Different Ages and Appendicular Lean Mass and Strength in Later Life: Results From the Berlin Aging Study II. J Gerontol A Biol Sci Med Sci. 2016;71(4):515–20. https://pubmed.ncbi.nlm.nih.gov/26442900/ Available from: Cited 2022 Jan 17 10.1093/gerona/glv171 26442900

[40] Rosique-Esteban N, Babio N, Díaz-López A, Romaguera D, Alfredo Martínez J, Sanchez VM, et al. Leisure-time physical activity at moderate and high intensity is associated with parameters of body composition, muscle strength and sarcopenia in aged adults with obesity and metabolic syndrome from the PREDIMED-Plus study. Clin Nutr. 2019;38(3):1324–31. https://pubmed.ncbi.nlm.nih.gov/29910068/ Available from: Cited 2022 Jan 17 10.1016/j.clnu.2018.05.023 29910068

[41] Choi BCK, Pak AWP. A catalog of biases in questionnaires. Prev Chronic Dis. 2005;2(1):A13. http://www.ncbi.nlm.nih.gov/pubmed/15670466. PMC1323316 15670466

[42] Tzeng PL, Lin CY, Lai TF, Huang WC, Pien E, Hsueh MC, et al. Daily lifestyle behaviors and risks of sarcopenia among older adults. Archives of Public Health 2020 78:1. 2020;78(1):1–8. https://archpublichealth.biomedcentral.com/articles/10.1186/s13690-020-00498-9 Available from: Cited 2021 Jul 21 10.1186/s13690-020-00498-9 PMC7653864 33292561

[43] Hayat SA, Luben R, Keevil VL, Moore S, Dalzell N, Bhaniani A (2014). Cohort profile: A prospective cohort study of objective physical and cognitive capability and visual health in an ageing population of men and women in Norfolk (EPIC-Norfolk 3). Int J Epidemiol.

[44] Day N, Oakes S, Luben R, Khaw KT, Bingham S, Welch A, et al. EPIC-Norfolk: study design and characteristics of the cohort. European Prospective Investigation of Cancer. Br J Cancer. 1999;80 Suppl 1:95–103. http://www.ncbi.nlm.nih.gov/pubmed/10466767 Available from: Cited 2016 Jul 13 10466767

[45] Ried-Larsen M, Brønd JC, Brage S, Hansen BH, Grydeland M, Andersen LB, et al. Mechanical and free living comparisons of four generations of the Actigraph activity monitor. International Journal of Behavioral Nutrition and Physical Activity. 2012;9(1):113. http://ijbnpa.biomedcentral.com/articles/. 10.1186/1479-5868-9-113. Available from: Cited 2019 Mar 25 10.1186/1479-5868-9-113 PMC3463450 22971175

[46] Robusto KM, Trost SG. Comparison of three generations of ActiGraph^TM^ activity monitors in children and adolescents. J Sports Sci. 2012;30(13):1429–35. http://www.ncbi.nlm.nih.gov/pubmed/22857599 Available from: Cited 2019 Mar 25 10.1080/02640414.2012.710761 PMC3458797 22857599

[47] Ojiambo R, Cuthill R, Budd H, Konstabel K, Casajús JA, González-Agüero A (2011). Impact of methodological decisions on accelerometer outcome variables in young children. Int J Obes (Lond).

[48] Edwardson CL, Gorely T. Epoch length and its effect on physical activity intensity. Med Sci Sports Exerc. 2010;42(5):928–34. Available from: Cited 2016 Nov 7. http://content.wkhealth.com/linkback/openurl?sid=WKPTLP:landingpage&an=00005768-201005000-00012. 10.1249/MSS.0b013e3181c301f5 19996997

[49] Troiano RP, Berrigan D, Dodd KW, Mâsse LC, Tilert T, McDowell M. Physical activity in the United States measured by accelerometer. Med Sci Sports Exerc. 2008;40(1):181–8. 10.1249/mss.0b013e31815a51b3. 10.1249/mss.0b013e31815a51b3 18091006

[50] Hall KS, Howe CA, Rana SR, Martin CL, Morey MC (2013). METs and accelerometry of walking in older adults: Standard versus measured energy cost. Med Sci Sports Exerc.

[51] Gorman E, Hanson HM, Yang PH, Khan KM, Liu-Ambrose & T, Ashe MC, et al. Accelerometry analysis of physical activity and sedentary behavior in older adults: a systematic review and data analysis. Eur Rev Aging Phys Act. 2014;11:35–49. http://www.pubmedcentral.nih.gov/articlerender.fcgi?artid=3990855&tool=pmcentrez&rendertype=abstract Available from: Cited 2014 Aug 18 10.1007/s11556-013-0132-x PMC3990855 24765212

[52] Hajna S, White T, Brage S, van Sluijs EMF, Westgate K, Jones AP, et al. Descriptive epidemiology of changes in objectively measured sedentary behaviour and physical activity: six-year follow-up of the EPIC-Norfolk cohort. International Journal of Behavioral Nutrition and Physical Activity. 2018;15(1):122. http://www.ncbi.nlm.nih.gov/pubmed/30482229 Available from: cited 2019 Mar 9 10.1186/s12966-018-0746-5 PMC6257971 30482229

[53] Berkemeyer K, Wijndaele K, White T, Cooper AJM, Luben R, Westgate K, et al. The descriptive epidemiology of accelerometer-measured physical activity in older adults. International Journal of Behavioral Nutrition and Physical Activity. 2016;13(1):2. http://www.ijbnpa.org/content/13/1/2 Available from: Cited 2017 Feb 5 10.1186/s12966-015-0316-z PMC4704380 26739758

[54] Kim TN, Park MS, Lee EJ, Chung HS, Yoo HJ, Kang HJ, et al. Comparisons of three different methods for defining sarcopenia: An aspect of cardiometabolic risk. Scientific Reports 2017 7:1. 2017;7(1):1–9. https://www.nature.com/articles/s41598-017-06831-7 Available from: Cited 2021 Nov 22 10.1038/s41598-017-06831-7 PMC5529503 28747657

[55] Kim KM, Jang HC, Lim S. Differences among skeletal muscle mass indices derived from height-, weight-, and body mass index-adjusted models in assessing sarcopenia. Korean J Intern Med. 2016;31(4):643–50. https://pubmed.ncbi.nlm.nih.gov/27334763/ Available from: Cited 2022 Jan 27 10.3904/kjim.2016.015 PMC4939509 27334763

[56] Smith L, Gardner B, Fisher A, Hamer M (2015). Patterns and correlates of physical activity behaviour over 10 years in older adults: prospective analyses from the English Longitudinal Study of Ageing. BMJ Open.

[57] Cepeda M, Koolhaas CM, van Rooij FJA, Tiemeier H, Guxens M, Franco OH, et al. Seasonality of physical activity, sedentary behavior, and sleep in a middle-aged and elderly population: The Rotterdam study. Maturitas. 2018;110:41–50. http://www.ncbi.nlm.nih.gov/pubmed/29563034 Available from: Cited 2019 Dec 5 10.1016/j.maturitas.2018.01.016 29563034

[58] Tin Tin S, Woodward A, Robinson E, Ameratunga S. Temporal, seasonal and weather effects on cycle volume: an ecological study. Environ Health. 2012;11(1):12. 10.1186/1476-069X-11-12. 10.1186/1476-069X-11-12 PMC3368741 22401535

[59] Stolwijk AM, Straatman H, Zielhuis GA. Studying seasonality by using sine and cosine functions in regression analysis. J Epidemiol Community Health (1978). 1999;53(4):235–8. http://www.ncbi.nlm.nih.gov/pubmed/10396550 Available from: Cited 2019 Dec 5 10.1136/jech.53.4.235 PMC1756865 10396550

[60] Steffl M, Bohannon RW, Sontakova L, Tufano JJ, Shiells K, Holmerova I. Relationship between sarcopenia and physical activity in older people: a systematic review and meta-analysis. Clin Interv Aging. 2017;12:835–45. http://www.ncbi.nlm.nih.gov/pubmed/28553092 Available from: Cited 2018 Sep 29 10.2147/CIA.S132940 PMC5441519 28553092

[61] Westbury LD, Dodds RM, Syddall HE, Baczynska AM, Shaw SC, Dennison EM, et al. Associations Between Objectively Measured Physical Activity, Body Composition and Sarcopenia: Findings from the Hertfordshire Sarcopenia Study (HSS). Calcif Tissue Int. 2018;103(3):237–45. https://pubmed.ncbi.nlm.nih.gov/29589060/ Available from: Cited 2022 Jan 17 10.1007/s00223-018-0413-5 PMC6049619 29589060

[62] Reid N, Healy GN, Gianoudis J, Formica M, Gardiner PA, Eakin EE, et al. Association of sitting time and breaks in sitting with muscle mass, strength, function, and inflammation in community-dwelling older adults. Osteoporos Int. 2018;29(6):1341–50. http://www.ncbi.nlm.nih.gov/pubmed/29479645 Available from: Cited 2020 Jun 25 10.1007/s00198-018-4428-6 29479645

[63] Szulc P, Duboeuf F, Marchand F, Delmas PD. Hormonal and lifestyle determinants of appendicular skeletal muscle mass in men: the MINOS study. Am J Clin Nutr. 2004;80(2):496–503. https://pubmed.ncbi.nlm.nih.gov/15277176/ Available from: cited 2022 Jan 17 10.1093/ajcn/80.2.496 15277176

[64] Kim SH, Kim TH, Hwang HJ. The relationship of physical activity (PA) and walking with sarcopenia in Korean males aged 60 years and older using the Fourth Korean National Health and Nutrition Examination Survey (KNHANES IV-2, 3), 2008–2009. Arch Gerontol Geriatr. 2013;56(3):472–7. https://pubmed.ncbi.nlm.nih.gov/23298535/ Available from: cited 2022 Jan 17 10.1016/j.archger.2012.12.009 23298535

[65] Negm AM, Lee J, Hamidian R, Jones CA, Khadaroo RG (2022). Management of Sarcopenia: A Network Meta-Analysis of Randomized Controlled Trials. J Am Med Dir Assoc.

[66] Gallagher D, Visser M, de Meersman RE, Sepúlveda D, Baumgartner RN, Pierson RN, et al. Appendicular skeletal muscle mass: effects of age, gender, and ethnicity. J Appl Physiol (1985). 1997;83(1):229–39. https://pubmed.ncbi.nlm.nih.gov/9216968/ Available from: Cited 2022 Jan 31 10.1152/jappl.1997.83.1.229 9216968

[67] Roh E, Choi KM (2020). Health Consequences of Sarcopenic Obesity: A Narrative Review. Front Endocrinol (Lausanne).

[68] Aunger JA, Doody P, Greig CA. Interventions targeting sedentary behavior in non-working older adults: a systematic review [Internet]. Vol. 116, Maturitas. Elsevier Ireland Ltd; 2018. p. 89–99. 10.1016/j.maturitas.2018.08.002 Available from: Cited 2020 Jul 21 10.1016/j.maturitas.2018.08.002 30244786

[69] Stockwell S, Schofield P, Fisher A, Firth J, Jackson SE, Stubbs B, et al. Digital behavior change interventions to promote physical activity and/or reduce sedentary behavior in older adults: A systematic review and meta-analysis. Exp Gerontol. 2019;120:68–87. https://linkinghub.elsevier.com/retrieve/pii/S0531556519300877 Available from: Cited 2019 Jun 12 10.1016/j.exger.2019.02.020 30836130

[70] Chase JAD. Interventions to increase physical activity among older adults: A meta-analysis. Vol. 55, Gerontologist. 2015:706–18. https://pubmed.ncbi.nlm.nih.gov/25298530/ Available from: Cited 2020 Jul 21 10.1093/geront/gnu090 PMC4542588 25298530

[71] Ross R, Chaput J-P, Giangregorio LM, Janssen I, Saunders TJ, Kho ME, Poitras VJ, Tomasone JR, El-Kotob R, McLaughlin EC, Duggan M, Carrier J, Carson V, Chastin SF, Latimer-Cheung AE, Chulak-Bozzer T, Faulkner G, Flood SM, Gazendam MK, Tremblay MS. Canadian 24-Hour Movement Guidelines for Adults aged 18–64 years and Adults aged 65 years or older: an integration of physical activity, sedentary behaviour, and sleep. Appl Physiol Nutr Metab. 2020;45(10 (Suppl. 2)):S57–102. 10.1139/apnm-2020-0467. 10.1139/apnm-2020-0467 33054332

[72] Brakenridge CJ, Healy GN, Sethi P, Carver A, Bellettiere J, Salim A (2021). Contrasting compositions of sitting, standing, stepping, and sleeping time: associations with glycaemic outcome by diabetes risk. Int J Behav Nutr Phys Act.

[73] NHS. Physical activity guidelines for older adults. 2021. https://www.nhs.uk/live-well/exercise/exercise-guidelines/physical-activity-guidelines-older-adults/ Available from: Cited 2023 Nov 9

[74] Mailey EL, Gothe NP, Wójcicki TR, Szabo AN, Olson EA, Mullen SP (2014). Influence of allowable interruption period on estimates of accelerometer wear time and sedentary time in older adults. J Aging Phys Act.

