# Supplementary material for: Prospective associations between changes in physical activity and sedentary time and subsequent lean muscle mass in older English adults: the EPIC-Norfolk cohort study
Source: Int J Behav Nutr Phys Act. 2024 Jan 26;21:10. doi: 10.1186/s12966-023-01547-6 (PMC10811887; doi:10.1186/s12966-023-01547-6)
Supplement: Supplementary file 1 — Additional file 1: Table S1. Participant characteristics for those included versus excluded. [file 12966_2023_1547_MOESM1_ESM.docx]

**Supplementary Table 1: Participant characteristics for those included versus excluded**

| **Characteristics** | | **Percent of Included participants (n=1,535) (%)** | **Percent of Excluded participants (n=77) (%)** |
| --- | --- | --- | --- |
| Sex | Male | 44.5 | 37.7 |
|  | Female | 55.5 | 62.3 |
| Ethnicity | White | 99.9 | 100 |
|  | Other | 0.1 | 0 |
| Occupational Classification | Professional | 9.0 | 8.0 |
|  | Manager | 43.4 | 40.3 |
|  | Skilled non-manual | 13.6 | 17.7 |
|  | Skilled manual | 21.4 | 17.7 |
|  | Semi-skilled | 10.6 | 16.3 |
|  | Non-skilled | 2.0 | 0 |
| Employed* | No | 76.8 | 77.3 |
|  | Yes | 23.2 | 22.7 |
| Further Education level | O-level or lower | 46.7 | 45.5 |
|  | A-level or higher | 53.3 | 54.6 |
| Smoking Status* | Current | 2.4 | 6.9 |
|  | Former | 45.3 | 47.2 |
|  | Never | 52.3 | 45.8 |
| History of Chronic Disease * | No | 84.4 | 81.8 |
|  | Yes | 15.6 | 18.2 |
| Body Mass Index (kg/m^2^)* | <25 | 36.8 | 28.5 |
|  | 25-<30 | 45.5 | 49.4 |
|  | 30-<35 | 13.9 | 18.2 |
|  | ≥35 | 3.8 | 3.9 |

*This table shows the percentage spread across categories of participant characteristics for those included versus excluded. The mean age of excluded individuals was 69.1, SD 6.3. Further education level categories include O level or lower (UK national qualification to age 16) vs A level or higher (UK national qualification over age 16).*
