# Supplementary material for: Prospective associations between changes in physical activity and sedentary time and subsequent lean muscle mass in older English adults: the EPIC-Norfolk cohort study
Source: Int J Behav Nutr Phys Act. 2024 Jan 26;21:10. doi: 10.1186/s12966-023-01547-6 (PMC10811887; doi:10.1186/s12966-023-01547-6)
Supplement: Supplementary file 2 — Additional file 2: Table S2. Exposure characteristics for alternative cut-points of LPA and MVPA. [file 12966_2023_1547_MOESM2_ESM.docx]

**Supplementary Table 2: Exposure characteristics for alternative cut-points of LPA and MVPA**

| **Exposure or Outcome** | **Baseline Mean (SD)** | | **Follow-up Mean (SD)** | | **Mean Annual Change (SD)** | |
| --- | --- | --- | --- | --- | --- | --- |
|  | **Male** | **Female** | **Male** | **Female** | **Male** | **Female** |
| **LPA (min/day)** | 280 (76.5) | 308.3 (73.9) | 244.0 (77.5) | 274.9 (76.6) | -6.8 (14.7) | -6.8 (15.9) |
| **MVPA (min/day)** | 26.2 (21.3) | 20.6 (17.2) | 20.7 (20.9) | 16.5 (16.5) | -1.0(4.6) | -0.8 (3.6) |

*This table shows the mean values of activity measures (alternative cut-points) at baseline and follow-up. Baseline measurements were undertaken between 2006-2011, and follow-up between 2012-2016. MVPA=moderate-to-vigorous activity (2020 cpm), LPA=light physical activity (100-2020 cpm), SD=standard deviation,*
