# Supplementary material for: Prospective associations between changes in physical activity and sedentary time and subsequent lean muscle mass in older English adults: the EPIC-Norfolk cohort study
Source: Int J Behav Nutr Phys Act. 2024 Jan 26;21:10. doi: 10.1186/s12966-023-01547-6 (PMC10811887; doi:10.1186/s12966-023-01547-6)
Supplement: Supplementary file 3 — Additional file 3: Table S3. Participation Rates of domain-specific activities. [file 12966_2023_1547_MOESM3_ESM.docx]

Supplementary Table 3: Participation Rates of domain-specific activities

| **Outcome** | **Baseline Rates (%)** | | **Follow-up Rates (%)** | |
| --- | --- | --- | --- | --- |
|  | **Male** | **Female** | **Male** | **Female** |
| **Walking** | 95.6 | 95.5 | 98.5 | 98.3 |
| **Cycling** | 29.2 | 20.2 | 25.2 | 18.0 |
| **Gardening** | 92.8 | 87.9 | 95.9 | 89.1 |
| **Housework** | 75.2 | 97.3 | 85.6 | 99.2 |
