# Supplementary material for: Prospective associations between changes in physical activity and sedentary time and subsequent lean muscle mass in older English adults: the EPIC-Norfolk cohort study
Source: Int J Behav Nutr Phys Act. 2024 Jan 26;21:10. doi: 10.1186/s12966-023-01547-6 (PMC10811887; doi:10.1186/s12966-023-01547-6)
Supplement: Supplementary file 5 — Additional file 5: Table S5. Association of change in physical activity and sedentary time with follow-up muscle mass. [file 12966_2023_1547_MOESM5_ESM.docx]

**Supplementary Table 5: Association of change in physical activity and sedentary time with follow-up muscle mass**

| **Change in Activity Measure** | **Model number** | **Follow-up** | | | | | | | | | | | |
| --- | --- | --- | --- | --- | --- | --- | --- | --- | --- | --- | --- | --- | --- |
|  |  | **Relative ALM (%)** | | | | **BMI-scaled ALM (kg/kg/m^2^)** | | | | **Height-scaled ALM** **(kg/m^2^)** | | | |
|  |  | **Men** | | **Women** | | **Men** | | **Women** | | **Men** | | **Women** | |
|  |  | **β** | **95%CI** | **β** | **95%CI** | **β** | **95%CI** | **β** | **95%CI** | **β** | **95%CI** | **β** | **95%CI** |
| **TPA (per 100 cpm/year)** | 1 | 3 | 2, 4 | 2 | 2, 3 | 0.08 | 0.04, 0.1 | 0.07 | 0.04, 0.1 | -0.2 | -0.5, 0.2 | -0.1 | -0.4, 0.2 |
|  | 2 | 2 | 2, 4 | 2 | 1, 3 | 0.09 | 0.05, 0.1 | 0.06 | 0.03, 0.9 | -0.001 | -0.005, 0.002 | -0.07 | -0.4, 0.3 |
|  |  |  |  |  |  |  |  |  |  |  |  |  |  |
| **MVPA (per min/day/year)** | 1 | 0.08 | 0.05, 0.1 | 0.05 | 0.03, 0.07 | 0.002 | 0.001, 0.004 | 0.001 | 0.0006, 0.002 | -0.001 | -0.01, 0.008 | 0.001 | -0.007, 0.009 |
|  | *2* | 0.08 | 0.05, 0.1 | 0.05 | 0.03, 0.07 | 0.002 | 0.001, 0.004 | 0.001 | 0.0005, 0.002 | -0.001 | -0.01, 0.008 | 0.002 | -0.006, 0.008 |
|  |  |  |  |  |  |  |  |  |  |  |  |  |  |
| **LPA (per min/day/year)** | 1 | 0.04 | 0.02, 0.06 | 0.04 | 0.03, 0.06 | 0.001 | 0.0003, 0.002 | 0.001 | 0.0006, 0.002 | -0.008 | -0.01, -0.001 | -0.007 | -0.01, -0.0007 |
|  | 2 | 0.04 | 0.02, 0.06 | 0.04 | 0.02, 0.06 | 0.001 | 0.0003, 0.002 | 0.001 | 0.0006, 0.002 | -0.007 | -0.01, 0.0007 | -0.006 | -0.01, 0.0004 |
|  |  |  |  |  |  |  |  |  |  |  |  |  |  |
| **Total sedentary time (per min/day/year)** | 1 | -0.03 | -0.05, -0.02 | -0.03 | -0.05, -0.02 | -0.001 | -0.002, -0.0005 | -0.001 | -0.001, -0.0006 | 0.005 | 0.0002, 0.01 | 0.003 | -0.002, 0.007 |
|  | 2 | -0.03 | -0.05,-0.02 | -0.03 | -0.05, -0.02 | -0.001 | -0.002, -0.0004 | -0.0009 | -0.001, -0.0005 | 0.004 | -0.0004, 0.009 | 0.002 | -0.003, 0.007 |
|  |  |  |  |  |  |  |  |  |  |  |  |  |  |
| **Prolonged ST bout time (per min/day/year)** | 1 | -0.02 | -0.03, -0.01 | -0.02 | -0.03, -0.01 | -0.0005 | -0.0009, -0.00003 | -0.0005 | -0.0009, -0.0002 | 0.003 | -0.0002, 0.007 | 0.004 | 0.0008, 0.008 |
|  | 2 | -0.02 | -0.03, -0.01 | -0.02 | -0.03, -0.01 | -0.0004 | -0.0009, -0.00003 | -0.0005 | -0.0008, -0.0001 | 0.003 | -0.0004, 0.007 | 0.004 | -0.0002, 0.007 |
|  |  |  |  |  |  |  |  |  |  |  |  |  |  |
| ***Sensitivity Analysis (MVPA defined as ≥2020cpm, LPA defined as 100-2019 cpm)*** | | | | | | | | | | | | | |
| **MVPA (per min/day/year)** | 1 | 0.1 | 0.07, 0.2 | 0.1 | 0.05, 0.2 | 0.004 | 0.002, 0.006 | 0.003 | 0.002, 0.005 | -0.0006 | -0.02, 0.01 | -0.007 | -0.03, 0.01 |
|  | *2* | 0.1 | 0.08, 0.2 | 0.1 | 0.05, 0.2 | 0.004 | 0.002, 0.006 | 0.003 | 0.001, 0.005 | -0.0007 | -0.02, 0.01 | -0.007 | -0.03, 0.01 |
|  |  |  |  |  |  |  |  |  |  |  |  |  |  |
| **LPA (per min/day/year)** | 1 | 0.03 | 0.02, 0.05 | 0.03 | 0.02, 0.05 | 0.001 | 0.0003, 0.002 | 0.0009 | 0.0005, 0.001 | -0.005 | -0.01, -0.0009 | -0.003 | -0.007, 0.002 |
|  | 2 | 0.03 | 0.01, 0.05 | 0.03 | 0.02, 0.04 | 0.0009 | 0.0003, 0.002 | 0.0008 | 0.0004, 0.001 | -0.005 | -0.01, 0.0005 | -0.002 | -0.007, 0.003 |
|  |  |  |  |  |  |  |  |  |  |  |  |  |  |

| **Change in Activity Measure** | **Model number** | **Follow-up** | | | | | | | | | | | |
| --- | --- | --- | --- | --- | --- | --- | --- | --- | --- | --- | --- | --- | --- |
|  |  | **Relative ALM (%)** | | | | **BMI-scaled ALM (kg/kg/m^2^)** | | | | **Height-scaled ALM (kg/m^2^)** | | | |
|  |  | **Men** | | **Women** | | **Men** | | **Women** | | **Men** | | **Women** | |
|  |  | **β** | **95%CI** | **β** | **95%CI** | **β** | **95%CI** | **β** | **95%CI** | **β** | **95%CI** | **β** | **95%CI** |
| **Walking time (per hour/week/year)** | 1 | 0.03 | -0.06, 0.1 | 0.05 | -0.04, 0.1 | 0.002 | -0.001, 0.006 | 0.001 | -0.002, 0.004 | 0.009 | -0.02, 0.03 | 0.003 | -0.03, 0.03 |
|  | 2 | 0.03 | -0.06, 0.1 | 0.04 | -0.05, 0.1 | 0.003 | -0.0004, 0.006 | 0.0009 | -0.002, 0.004 | 0.009 | -0.02, 0.04 | 0.0005 | -0.03, 0.03 |
|  |  |  |  |  |  |  |  |  |  |  |  |  |  |
| **Cycling time (per hour/week/year)** | 1 | 0.2 | -0.07, 0.5 | 0.9 | 0.4, 1.5 | 0.009 | -0.003, 0.02 | 0.03 | 0.008, 0.04 | 0.01 | -0.08, 0.1 | 0.04 | -0.1, 0.2 |
|  | *2* | 0.2 | -0.1, 0.5 | 0.9 | 0.4, 1.5 | 0.01 | -0.001, 0.02 | 0.03 | 0.008, 0.04 | -0.01 | -0.1, 0.09 | 0.02 | -0.2, 0.2 |
|  |  |  |  |  |  |  |  |  |  |  |  |  |  |
| **Gardening time (per hour/week/year)** | 1 | 0.2 | 0.02, 0.3 | 0.2 | 0.03, 0.2 | 0.006 | -0.00006, 0.01 | 0.003 | 0.0006, 0.007 | -0.008 | -0.05, 0.03 | 0.006 | -0.03, 0.04 |
|  | 2 | 0.1 | -0.02, 0.2 | 0.2 | 0.04, 0.3 | 0.003 | -0.002, 0.007 | 0.004 | 0.0009, 0.008 | -0.009 | -0.05, 0.03 | 0.0003 | -0.04, 0.04 |
|  |  |  |  |  |  |  |  |  |  |  |  |  |  |
| **Housework (per hour/week/year)** | 1 | -0.07 | -0.2, 0.04 | 0.02 | -0.04, 0.08 | -0.004 | -0.008, 0.001 | -0.0003 | -0.002, 0.002 | 0.003 | -0.03, 0.04 | 0.02 | 0.0004, 0.04 |
|  | 2 | -0.06 | -0.2, 0.05 | 0.02 | -0.04, 0.08 | -0.002 | -0.006, 0.002 | -0.0008 | -0.002, 0.002 | 0.004 | -0.03, 0.04 | 0.02 | 0.0007, 0.04 |
|  |  |  |  |  |  |  |  |  |  |  |  |  |  |

*This table shows the associations of change in physical activity and sedentary time (baseline to follow-up) with follow-up muscle measures (****n=1436)****. Coding of season was using sine and cosine functions; spring = sin (2*π* day of year /365.25), winter = cos (2 *π* day of year/365.25)]. The main analysis used activity variable cut-points of ≥809 cutpoint for MVPA and 100-808 cutpoint for LPA. The sensitivity analyses shown here used ≥2020cpm for MVPA and 100-2019cpm for LPA.*

*TPA=total physical activity, MVPA=moderate-to-vigorous activity, LPA=light physical activity,* ALM = appendicular lean muscle mass, BMI=body mass index, h=height

*Model 1 was adjusted for season and wear time at baseline and follow-up, and baseline activity measure, age and sex*

*Model 2 was the same as Model 1 plus mutually adjusted for potential socioeconomic and environmental confounders (job status, smoking status, occupational class, chronic disease status, and household financial status).*
